# Gadoxetic acid-enhanced magnetic resonance imaging significantly influences the clinical course in patients with colorectal liver metastases

**DOI:** 10.1186/s12880-018-0289-x

**Published:** 2018-11-15

**Authors:** B. G. Sibinga Mulder, K. Visser, S. Feshtali, A. L. Vahrmeijer, R. J. Swijnenburg, H. H. Hartgrink, R. van den Boom, M. C. Burgmans, J. S. D. Mieog

**Affiliations:** 1 Department of Surgery, Leiden University Medical Center, P.O. Box 9600, Leiden, 2300 RC The Netherlands; 2 Department of Radiology, Leiden University Medical Center, P.O. Box 9600, Leiden, 2300 RC The Netherlands

**Keywords:** Colorectal liver metastases, Primovist MRI, Treatment proposition

## Abstract

**Background:**

Gadoxetic acid (Primovist™)-enhanced magnetic resonance imaging (P-MRI) scans have higher accuracy and increased detection of small colorectal liver metastases (CRLM) compared to CT scans or conventional MRI scans. But, P-MRI scans are still inconsistently acquired in the diagnostic work up of patients with CRLM. The aim of this study was to determine the influence of P-MRI scans on treatment plan proposition and subsequently the clinical course of the patient.

**Methods:**

Eighty-three consecutive patients with potentially resectable CRLM based on a conventional CT scan underwent P-MRI scanning prior to treatment. Treatment plans proposed by the multidisciplinary team were compared before and after P-MRI scanning and related to the final treatment and diagnosis, the accuracy for the CT scan and P-MRI scan was calculated.

**Results:**

P-MRI scans led to a change of treatment in 15 patients (18%) and alteration of extensiveness of local therapy in another 17 patients (20%). All changes were justified leading to an accuracy of 93% for treatment proposition based on P-MRI scan, compared to an accuracy of 75% for the CT scan.

**Conclusions:**

P-MRI scans provide additional information that can aid in proposing the most suitable treatment for patients with CRLM and might prevent short-term reintervention.

**Electronic supplementary material:**

The online version of this article (10.1186/s12880-018-0289-x) contains supplementary material, which is available to authorized users.

## Background

Liver metastases arise in 50–65% of patients with colorectal cancer [1, 2]. Long-term survival can be achieved with surgical resection or local ablation therapy in patients with resectable colorectal liver metastases (CRLM).

Imaging plays a principal role in staging of CRLM. Nowadays, the most used imaging modalities are contrast-enhanced computed tomography (CT) or multi-detector row CT (MDCT). Both are limited in the detection of nodal involvement and characterization of small liver lesions compared to magnetic resonance imaging (MRI) scans [3]. A CT scan has a sensitivity and specificity of 68 and 94%, respectively, and a gadolinium contrast-enhanced MRI scan of 90 and 87%, respectively [4]. Different contrast agents can be administered for contrast-enhanced hepatic MRI scans, like gadoxetic acid, also called Primovist™ (P-MRI) (Bayer AG, Germany) [5, 6]. The reported sensitivity of P-MRI for the detection of CRLM is 87–100% and the specificity is approximately 95% [7–9]. However, CT is still the most frequently used to assess liver involvement and plan surgical resection (Dutch Oncoline guidelines: CRC). P-MRI is not routinely acquired prior to liver resection in most centers [8, 10]. In addition, literature is lacking about the direct effect on treatment strategy in a clinical setting, including local therapy, systemic therapy or conservative treatment in a larger patient cohort. Therefore, there is still no convincing evidence whether P-MRI scans should play a more prominent role in the diagnostic process of patients with potential treatable CRLM and should be standardly acquired. The aim of this study was to determine the impact of P-MRI scans on treatment plan proposition and subsequently the clinical course of patients with potentially treatable CRLM, based on preoperative CT scans.

## Methods

The study was designed as a retrospective cohort study in which consecutive patients were included. The study was approved by the scientific review board of the Department of Radiology of the Leiden University Medical Center (LUMC) in 2017. Patient confidentiality was guaranteed using anonymized data and radiologic images, and all data was entered into an encrypted and secured database.

### Patients

**Fig. 1:**
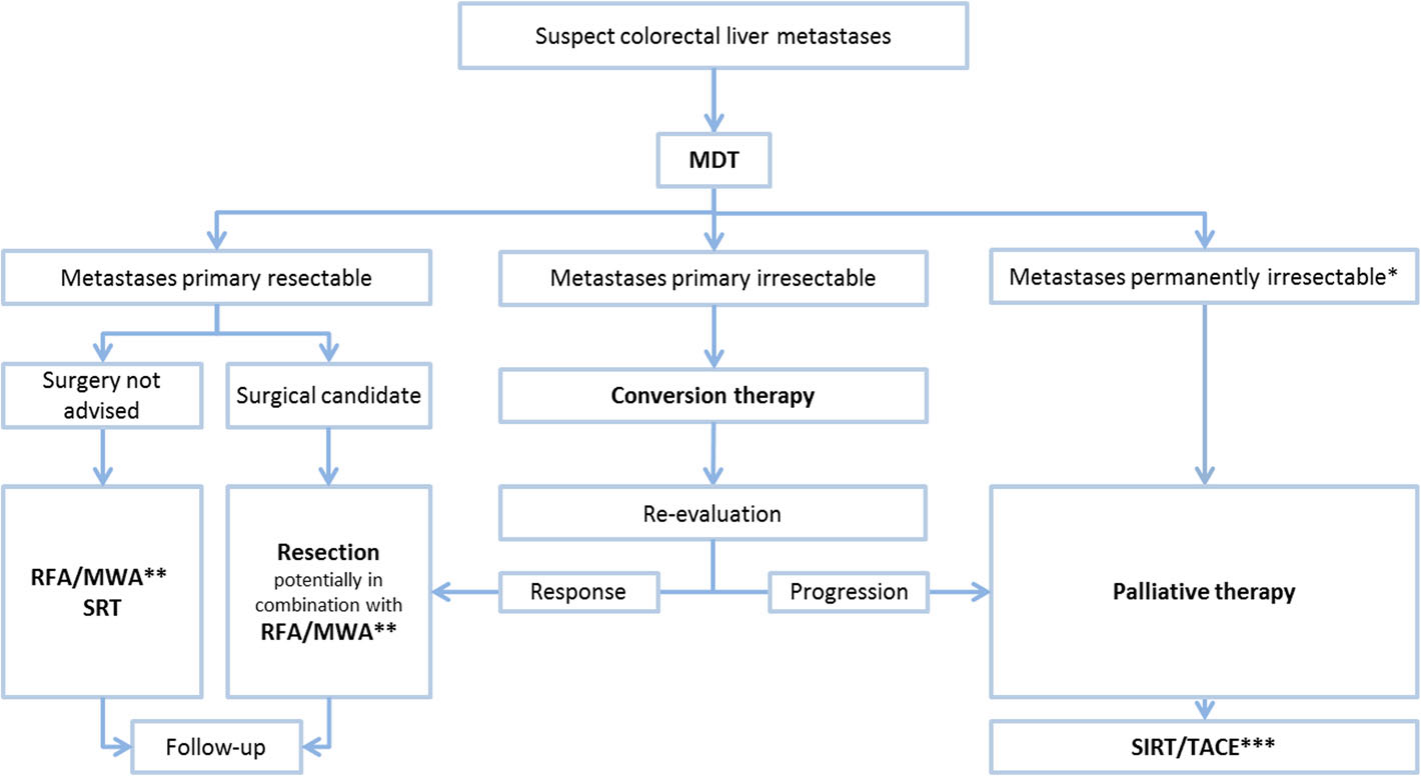
Standard operation procedure concerning the loco regional treatment of CRLM in the Leiden University Medical Center

All patients with potential CRLM were discussed by a multidisciplinary team (MDT) of hepatobiliary surgeons, medical oncologists and (interventional) radiologists. In Fig. 1 a flowchart is presented illustrating the standard operation procedure concerning the loco regional treatment of CRLM in the LUMC. Patients presented at the MDT meeting between July 2014 and August 2017 with primary resectable or primary irresectable (but potentially resectable after conversion therapy) CRLM based on the contrast enhanced (CE)-CT scan, of whom a P-MRI was obtained, were included in this study. Patients with permanent irresectable CRLM were not included. Patients who were previously treated for their CRLM with local therapy or systemic chemotherapy were also included. Patients in whom the time interval between acquirement of the CT scan and P-MRI or P-MRI and treatment was > 2 months were excluded. Age, sex, localization of CRC, number of comorbidities, previous surgery and/or systemic therapy for CRC, and previous local therapy and/or systemic therapy for CRLM were noted.

### CT scan

All CT scans included an arterial phase scan and a portal venous phase scan, using slices of 5 mm or less for reconstructive images. Contrast-enhance CT scans obtained in the LUMC were performed on a 16-slice spiral CT (Aquillion-16, Toshiba, Tokyo, Japan) using the following scanning parameters: 16 × 1 mm scanning, 120 KV, rotation 0.5 s, contrast Ultravist 370 (dose weight depended: for a standard patient (75 kg) 120 ml was injected. Based on weight categories 46–60 kg, 60–80 kg, 80–100 kg, 100+ kg 20% more or less of the dose was administered) with a delay of 75 s for portal venous phase. Further parameters of CT scanning: the current modulation for the tube varying is from 10 to 500 mA. The pitch factor is 0.8125. Noise index with standard deviation of 10 is used. An Adaptive Iterative Dose Reduction (AIDR) 3D is used as reconstruction algorithm. The reconstruction kernel is FC 18 (soft tissue filter). The CT scans were scored for the presence, the number, location and size of all liver metastases. All scans were evaluated by one of four radiologists from the LUMC, all with at least 5 years of experience in liver imaging.

### P-MRI scan

All P-MRI scans were performed in the LUMC on a Philips Ingenia 1.5 or 3.0 T. Gadoxetic acid was the intravenously administered contrast medium (10 ml Primovist). MRI sequences included transveral and coronal T2 TSE (TE 80 and 250), transversal T1FFE mDIXON, diffusion weighted imaging at b0–10–500–1000, dynamic multiphase contrast-enhanced T1FFE images, including 20 min post-contrast images. See Additional file 1: Table S1 for additional information about the used sequences. P-MRIs were read by one of four dedicated abdominal radiologist, each with at least x years of experience after board certification in radiology. Readers were not blinded for clinical information or prior medical imaging.

### Study design

All patients were discussed by the MDT, which is a weekly meeting during which all patients who are potentially operable are discussed. As stated before, Fig. 1 gives an overview of all possible treatment options in the LUMC. During the first MDT meeting a treatment plan was proposed based on the CT scan and classified as [1]: local therapy, consisting of either resection, percutaneous ablation or resection + ablation by radiofrequency ablation or microwave ablation [2], conversion therapy with intent of local therapy or [3] follow-up in case of suspicion of benign lesion(s). Of note, patient with irresectable disease were excluded. Subsequently, an P-MRI scan was made and a treatment plan was proposed during the second MDT meeting, potentially changing the previously proposed treatment plan [1]: local therapy [2], adjustment of the local therapy, in case the extensiveness of the local therapy was altered [3], conversion therapy [4], palliation, in case of diffuse disseminated disease resulting in less than 20% of healthy liver parenchyma, or extrahepatic disease (EHD) or [5] follow-up.

Finally, the proposed treatment plan based on the P-MRI scan was compared to the actual performed treatment.

For clarification, ‘conversion therapy’ is administered with the purpose of reverting the disease from irresectable to resectable. Conversion therapy was mostly a combination of capecitabine with oxaliplatin (CAPOX) usually given in 3 week cycles with a maximum of eight cycles total. ‘Palliation’ is applied if resection is not considered a (future) option.

In case the patients underwent surgery, the liver was assessed with intra-operative ultrasound by a radiologist. Additionally, in some patients near-infrared fluorescence (NIRF) imaging, using indocyanine green, was performed for the detection of occult liver metastases [11].

### Follow-up

In case the liver lesions were resected, standard pathological assessment of the lesions was performed. The pathological assessment was used as reference. Follow-up of the liver occurred every 4 months with CE-CT scan and CEA serum level measurements. In case new intrahepatic lesions were identified during the first follow-up visit, these lesions were probably already present during the procedure and were therefore missed on the P-MRI scan. During the follow-up process of the patients whose lesions were defined as benign, it became clear if these lesions were indeed not CRLM. A minority of patients were referred back to the external hospital for follow-up.

### Statistical analysis

Data were analyzed using a statistical software program (SPSS, version 23.0). The change in treatment proposition between MDT meeting 1 and 2 was evaluated using an independent samples *t*-test with standard error and 95% confidence interval. Furthermore, an independent samples *t*-test was performed for 1) comparison of the interval between the date of P-MRI scan and date of actual treatment between the patients who underwent local therapy without adjustment of the local therapy and with adjustment, and 2) for the change in treatment proposition between the group with CT-scan acquired in an external hospital and in the LUMC. The statistical results were considered to indicate significance if the *P*-value was less than 0.05.

## Results

### Patients

In total, 83 patients were included in the study cohort. Patient and treatment characteristics are summed in Table 1.

**Table 1:** Patient and treatment characteristics

	*N* = 83
Age at time of CT scan; mean ± SD	64.8 ± 10.7
Sex, male; n (%)	56 (68)
Synchronous CRLM; n (%)	44 (53)
Comorbidities; n (%)	
0	36 (43)
1	28 (34)
≥ 2	19 (23)
Previous CRLM surgery, n (%)	18 (22)
CT scan made in LUMC, n (%)	32 (39)
Days between; mean ± SD	
CT and P-MRI	29.0 ± 17.1
Days between P-MRI and treatment	28.0 ± 15.8
Actual treatment; n (%)	
Local	50 (60)
Neo-adjuvant chemotherapy	15 (18)
Palliation	10 (12)
Follow-up	8 (10)

### Treatment proposition

The type of treatment was changed in 15 patients (18%) due to the P-MRI scans, all these changes were in concordance with the actual treatment performed.

The intended extensiveness of the local therapy was altered in another 17 patients (20%) due to the P-MRI scan: in 11 patients more malignant lesions were identified with P-MRI (mean number additional lesions: 1.6 range; 1–3) (mean size additional lesions: 6.7 mm; range 2–16 mm), the additional identified lesions were located peripheral, superficial and central in the liver. In six patients less lesions were defined as malignant (mean number: 2.0 range; 1–6). These lesions were either considered as cysts (*n* = 3), haemangiomas (*n* = 5), steatosis (*n* = 2) or could not be retrieved (*n* = 2) on the P-MRI scan.

Together the treatment plan proposition was altered in 32 patients (38%; standard error of the mean: 5%; 95% confidence interval: 28–49%). The treatment plan of the remaining 51 patients (61%) was not altered. Table 2 gives an overview of the changes in treatment plan.

**Table 2:** Comparison of the treatment plans proposed during MDT 1 and MDT 2

	MDT 2						
MDT 1		Local	Local therapy adjustment	Neoadjuvant	Palliation	Follow-up	Total
	Local	39	*17*	**5**	**3**	**4**	68
	Neoadjuvant	0	0	9	**1**	**1**	11
	Follow-up	**1**	0	0	0	3	4
	Total	40	17	14	4	8	83

Changes in treatment plan are shown in bold. Extensiveness adjustment of local therapy in italic

### Actual treatment

In 56 patients the intended treatment based on the P-MRI scan was local therapy and the extensiveness of the local therapy was altered during the treatment in 10 patients, either more lesions (in six patients) or less lesions (in 4 four patients) were detected and resected during surgery. For detection of lesions ultrasound and NIRF imaging could be used. The additional lesions were biopsied for histological confirmation. The patients with lesions that could not be retrieved did not develop CRLM during the follow-up period, so these lesions were considered false-positive.

In five patients, who were deemed resectable on both CT and MRI, no resection or ablation was performed because a too diffuse disease (*n* = 3) or too extensive tumour burden (*n* = 2) was encountered during surgical exploration. They were palliated instead.

In one patient the proposed treatment based on the CT-scan and P-MRI was local therapy but eventually this patient was not fit for surgery, therefore, he was palliated.

The extensiveness of local therapy of the other 40 patients was not altered, they were treated within a mean of 29.0 days (SD 12.6 days) after the P-MRI scan was obtained, which was not significantly different from the 15 patients with adjusted local therapy (28.3 days (SD 20.7 days)) (*P*-value = 0.904).

In addition, there was no significant difference in change of treatment plan between patients whose CT scans were from external hospitals (31% change of treatment) and patients whose CT scans were from the LUMC (41% change of treatment) (*p*-value = 0.48).

**Fig. 2:**
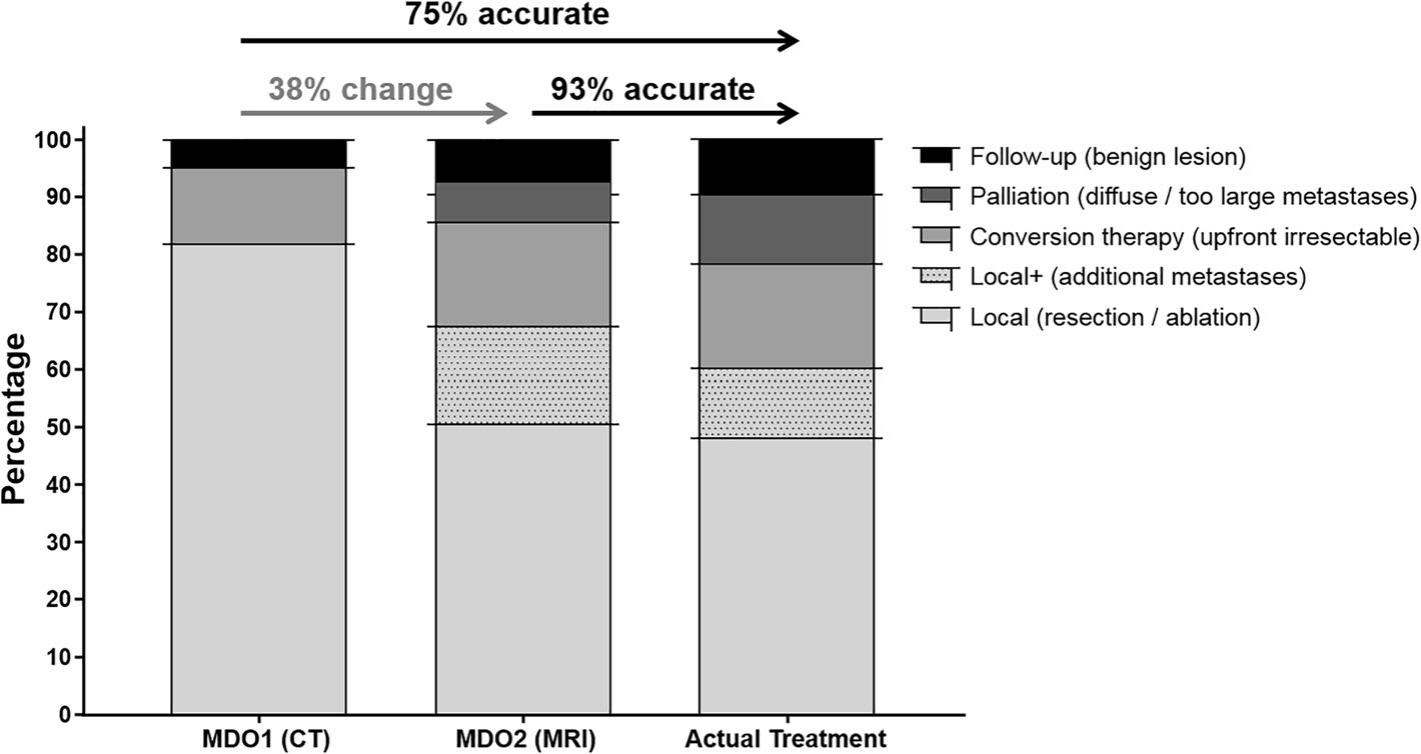
Changes in treatment plan and actual treatment in percentages

An overview of the changes in treatment plan proposition during MDT meeting 1 and 2, and the actual treatment performed is given in Fig. 2.

### Follow-up

All patients had a follow-up period of at least 3 months, median follow-up period was 14.4 months (1.38–41.7). Fifteen (18%) patients developed new intrahepatic CRLM within a median time of 5.3 months (0.8–14.0) after local treatment, in three of these patients the new intrahepatic CRLM occurred within 4 months after local therapy. Of the eight patients with lesions deemed benign, the median follow-up period was 22.0 months (4.1–41.2). One patient developed diffuse CRLM within 6 months. This patient presented with one lesion that couldn’t be characterized on the CT scan and was assessed as a hemorrhagic-cyst on the P-MRI, but eventually turned out to be a metastasis.

### Accuracy

The treatment plan proposition based on the P-MRI was accurate in 77 patients (93%). In five patients an too extensive disease was encountered during surgery and in one patient a CRLM was characterized as a hemorrhagic-cyst on the P-MRI scan. The treatment plan proposition based on the CT scan of these six patients was the same as the P-MRI. The 15 changes of treatment type due to P-MRI findings were all correct, so the accuracy of the CT scan for treatment plan proposition was accurate in 62 patients (75%).

## Discussion

Our study focusses on the effect of the P-MRI on the treatment plan proposition and clinical course in patient with potential resectable CRLM based on CT scans. The initial treatment plan proposal based on the CT scan was changed in 38% of the patients due to the findings on P-MRI scans. Compared to the final treatment, the proposition based on the P-MRI scan was accurate in 93% of the patients, compared to 75% of the propositions based on CT scans.

The diagnostic accuracy of the P-MRI scans in patients with CRLM and also the impact on surgical strategy has been previously described: in a study of Sofue et al., the surgical strategy changed in 33% of the patients due to the P-MRI [12]. The recent study of Jhaveri et al. describes changes in surgical strategy due to P-MRI scans in 45% of the patients receiving neoadjuvant therapy [13]. In contrast, Kang et al. also investigated the changes in surgical strategy, but only changed the surgery in 3% of the patients [14]. Only Vreugdenburg et al. also determined the impact of P-MRI scans on patient management, but only four of the 13 included studies investigated patient outcome, mainly focusing on surgical strategy instead of treatment strategy [9].

Our single center study emphasizes not only the impact of P-MRI scans on surgical strategy but on the entire treatment plan proposition. The implementation of obtaining a P-MRI scan in the diagnostic work-up of patients has several positive effects on the clinical course of the patients. The most important consequence is the prevention of unnecessary surgeries due to better differentiation between malignancies and benign lesions on P-MRI scans. A second advantage, which is also nicely demonstrated by the study of Knowles et al. is the more accurate staging based on P-MRI scans prior to conversion chemotherapy, thereby reducing intra-hepatic recurrence and avoid repeated hepatectomy [15]. In our study more patients were treated with conversion therapy prior to surgery due to new findings on the P-MRI scan. Furthermore, in other patients the intended local therapy was extended due to the additional identification of small lesions with P-MRI, resulting in the prevention of short-term reinterventions for new intrahepatic lesion development after treatment. P-MRI scanning aids in surgical planning, surgeons and/or interventional radiologist are preoperatively better prepared in comparison to CT scan alone, which might result in more curative resections and less new intrahepatic CRLM formation quickly after surgery. However, these lesions might be detected during local treatment anyway with ultrasound or other additional techniques as NIRF-guided surgery, which has the potential to detect additional small superficial lesion in approximately 15% of the patients [11, 16]. All current studies still advocate repeated therapy or resection to treat all CRLM, including recurrent CRLM, to improve survival rate [17, 18]. In addition, liver parenchyma sparing therapy improves 5-year survival rates, because repeated therapy can be applied if desired [19]. Therefore, accurate imaging can aid in proposing the most suitable treatment strategy, prevent reinterventions and treat as liver parenchyma sparing as possible. In combination with supplementary techniques, as NIRF guided surgery and intraoperative ultrasound, not only detection of CRLM will increase, also radical resection rates will improve and reinterventions can be prevented, all resulting in improved patient outcome. Therefore, future studies should also focus on the bundling of all pre- and intra-operative techniques.

Our study has several limitations. Due to the retrospective study design, decision making was retrospectively collected, however, consecutive patients were included and image and treatment plan evaluation during the MDT meetings was performed in real time and well-documented in the electronic patient charts. This resulted also a non-standardized assessment of the scans by four different radiologists. Differences between CT and MRI scan could be explained due to the differences between readers. Furthermore, readers were not blinded for the CT scans during P-MRI assessment. However, this resulted in a realistic representation of the “everyday practice”. Additionally, the follow-up scans were CT scans and not P-MRI scans because this is performed according to the current national guidelines. Time-interval between obtaining the P-MRI scan and the actual treatment might be of influence. But, we found no significant differences for both the origin of acquirement of CT scans or for the time-interval. Sensitivity and specificity could not be calculated based on our study results without introducing bias. Moreover, sensitivity and specificity have been examined extensively in multiple studies, and will be of no additional value. Finally, we can interpret our results only by looking at the patient’s clinical course, based on which the treatment plan proposition using P-MRI had an accuracy of 93%. However, we have no proof that these treatment propositions were superior to the treatment propositions based on the CT scans, especially, those in whom the patients were administered conversion therapy instead of upfront local therapy, as described above.

To conclude and most importantly, our study is a display of the clinical decision making in a Dutch academic center, underlining the added value of a P-MRI scan in the entire treatment process of CRLM, improving cancer care and clinical outcome for these patients. In which we showed that P-MRI scans provide additional information that can aid in proposing the most suitable treatment and might prevent short-term reintervention of patients with CRLM. Incorporating an P-MRI scan in the work-up of CRLM patients can improve treatment proposition and clinical outcome compared to CT scanning alone.

## Additional file

Additional file 1: MRI scan protocols 1.5 Tesla and 3 Tesla Ingenia. Both scanning protocols of 1.5 T and 3 T Ingenia are displayed in a table in the order of acquiring the sequences. (DOCX 20 kb)

## Abbreviations

CE-CT: Contrast enhanced CT
CRLM: Colorectal liver metastases
CT: Computed tomography
EHD: Extrahepatic disease
MDCT: Multi-detector row CT
MDT: Multidisciplinary team
MRI: Magnetic resonance imaging
NIRF: Near-infrared fluorescence
P-MRI: Primovist MRI
