# Supplementary material for: Gadoxetic acid-enhanced magnetic resonance imaging significantly influences the clinical course in patients with colorectal liver metastases
Source: BMC Med Imaging. 2018 Nov 15;18:44. doi: 10.1186/s12880-018-0289-x (PMC6238306; doi:10.1186/s12880-018-0289-x)
Supplement: Supplementary file 1 — MRI scan protocols 1.5 Tesla and 3 Tesla Ingenia. Both scanning protocols of 1.5 T and 3 T Ingenia are displayed in a table in the order of acquiring the sequences. (DOCX 20 kb) [file 12880_2018_289_MOESM1_ESM.docx]

**Additional file**

**Gadoxetic acid-enhanced magnetic resonance imaging significantly influences the clinical course in patients with colorectal liver metastases**

B.G. Sibinga Mulder^1^, B.S., K. Visser^1^, B.S., S. Feshtali^2^, MD, A.L. Vahrmeijer^1^, MD, PhD, R.J. Swijnenburg^1^, MD, PhD, H.H. Hartgrink^1^, MD, PhD, R. van den Boom^2^, MD, PhD, M.C. Burgmans^2^, MD, PhD, J.S.D. Mieog^1^, MD, PhD.

*^1^ Department of Surgery, Leiden University Medical Center, the Netherlands*

*^2^ Department of Radiology, Leiden University Medical Center, the Netherlands*

**MRI scan protocols 1.5 Tesla and 3 Tesla Ingenia**

Both scanning protocols of 1.5T and 3T Ingenia are displayed in a table in the order of acquiring the sequences. During all scans bolus tracking was used. The timing of the post-contrast sequences were 20s, 1 min, 2min and 20min after contrast administration. For hepatobiliary T1w sequence a flip angle of 10 degrees was used.

**1.5T:**

| **Name** | **Technique** | **Orientation** | **FOV** | **Slices** | **Thickness** | **Voxel size** | **Scan time** |
| --- | --- | --- | --- | --- | --- | --- | --- |
| SURVEY | T1FFE | MST |  |  |  |  | 0:15 |
| T2 TSE COR | TSE | COR | 350x330 | 30 | 6.0 | 1.4 x 1.6 | 0.17 |
| mDIXON | T1FFE | TRA | 400x350 | 100 | 2.5 | 1.8 x 1.9 | 0:19 |
| DYNAMIC | T1FFE | TRA | 400x350 | 125 | 2.0 | 2.0 x 2.0 | 4 x 0:18 |
| T2 TSE TE80 | TSE | TRA | 400x350 | 38 | 6.0 | 1.4 x 1.6 | 0:17 |
| T2 TFE TE250 | TFE | TRA | 400x350 | 38 | 6.0 | 1.4 x 1.6 | 2 x 00:13 |
| DWI 0-10-500-1000 | SE-EPI | TRA | 400x350 | 38 | 6.0 | 3.0 x 3.0 | 4:45 |
| DYNAMIC late | T1FFE | TRA | 400x350 | 125 | 2.0 | 2.0 x 2.0 | 0:18 |

**3T Ingenia:**

| **Name** | **Technique** | **Orientation** | **FOV** | **Slices** | **Thickness** | **Voxel size** | **Scan time** |
| --- | --- | --- | --- | --- | --- | --- | --- |
| SURVEY | T1FFE | MST |  |  |  |  | 0:15 |
| T2 TSE TE80 COR | TSE | COR | 348 | 30 | 6.0 | 1.30 x 1.62 | 2 x 0:15 |
| mDIXON | T1FFE | TRA | 349 | 131 | 1.75 | 1.59 x 1.71 | 0:19 |
| DYNAMIC | T1FFE | TRA | 349 | 131 | 1.75 | 1.75 x 1.74 | 4 x 0:15 |
| T2 TSE TE80 | TSE | TRA | 348 | 34 | 6.0 | 1.30 x 1.62 | 1:42 |
| T2 TFE TE250 | TFE | TRA | 348 | 34 | 6.0 | 1.30 x 1.60 | 1:42 |
| DWI | SE-EPI | TRA | 352 | 34 | 6.0 | 3.03 x 3.03 | 4:36 |
| DYNAMIC late | T1FFE | TRA | 349 | 131 | 1.75 | 1.75 x 1.74 | 0:15 |
